# Sex Differences in Multiple Myeloma Biology but not Clinical Outcomes: Results from 3894 Patients in the Myeloma XI Trial

**DOI:** 10.1016/j.clml.2021.04.013

**Published:** 2021-10

**Authors:** Sarah Bird, David Cairns, Tom Menzies, Kevin Boyd, Faith Davies, Gordon Cook, Mark Drayson, Walter Gregory, Matthew Jenner, John Jones, Martin Kaiser, Roger Owen, Graham Jackson, Gareth Morgan, Charlotte Pawlyn

**Affiliations:** 1 The Institute of Cancer Research, London; 2 The Royal Marsden Hospital, London; 3 Clinical Trials Research Unit, Leeds Institute of Clinical Trials Research, University of Leeds, Leeds; 4 Perlmutter Cancer Center, NY Langone Health, New York, USA; 5 Clinical Immunology, University of Birmingham, Birmingham; 6 University Hospital Southampton NHS Foundation Trust, Southampton; 7 St James's University Hospital, Leeds; 8 Department of Haematology, Newcastle University, Newcastle

**Keywords:** myeloma, Sex differences, Clinical trials, Immunomodulatory drugs+

## Abstract

**Background:**

Sex differences in the incidence and outcomes of several cancers are well established. Multiple myeloma (MM) is a malignant plasma cell dyscrasia accounting for 2% of all new cancer cases in the UK. There is a clear sex disparity in MM incidence, with 57% of cases in males and 43% in females. The mechanisms behind this are not well understood and the impact of sex on patient outcomes has not been thoroughly explored.

**Patients and Methods:**

We investigated the association of sex with baseline disease characteristics and outcome in 3894 patients recruited to the phase III UK NCRI Myeloma XI trial, in which treatment exposure to lenalidomide predominated.

**Results:**

Females were significantly more likely to have the molecular lesions t(14;16) and del(17p) and were more likely to meet the cytogenetic classification of high-risk (HiR) or ultra-high-risk disease (UHiR). There was no difference in progression-free survival (PFS) or overall survival (OS) between the sexes in the overall population.

**Conclusion:**

Our data suggest that the genetic lesions involved in the initiation and progression of MM may be different between the sexes. Although females were more likely to have the poor prognosis lesions t(14;16) and del(17p), and were more likely to be assessed as having HiR or UHiR disease, this was not associated with reduced PFS or OS. In female patients the trial treatment may have been able to overcome some of the adverse effects of high-risk cytogenetic lesions.

**MicroAbstract**

Multiple myeloma (MM) is more common in males compared to females but the reasons behind this are not well understood and the impact of sex on patient outcomes is unclear. This study demonstrates fundamental differences in genetic lesions underlying the biology of MM between males and females. However, we found that progression-free survival and overall survival were the same in both sexes.

## Introduction

Sex differences in the incidence and outcomes of several cancers are now widely established^1^, ^2^, ^3^. Multiple myeloma (MM) is a malignancy of plasma cells and is the second most common haematological cancer^4^. MM is more common in males than females; in the UK the age-standardised incidence rate of MM is 11.6 per 100,000 per year in males and 7.3 per 100,000 per year in females^4^. The mechanisms driving this difference are poorly understood. In addition, the effect of sex on MM outcome has not been thoroughly explored.

The causes of sex disparities in cancer are not well-understood and multiple factors may be involved. Sex differences may influence cancer susceptibility at the genetic level and the development of cancer may be affected by sex hormones^5^. Furthermore, biological differences may affect response to therapeutic agents^5^. For example, it has been shown that the clearance, half-life and side effect profiles of many anticancer drugs are different between the sexes^5^.

MM develops from plasma cell clones that have accumulated a series of genetic lesions leading to a survival advantage^6^. In approximately half of patients the initiating genetic event is hyperdiploidy, characterised by trisomies of chromosomes 3, 5, 7, 9, 11, 15, 19 and 21^6^^,^ ^7^. The remaining half of patients usually have translocations affecting the immunoglobulin heavy chain gene (*IGH*) on 14q32 and a partner chromosome (frequently chromosomes 4, 6, 11, 16 or 20)^6^. The translocation event brings a partner oncogene under the influence of the *IGH* promoter/enhancer region which leads to upregulation of oncogene expression^6^. These initial events are followed by secondary genetic events that drive malignant progression and include gain of genetic material (e.g. gain(1q21)), loss of genetic material (e.g. del(13)q, del(17p)) and epigenetic modifications^6^. Several of these genetic lesions have been associated with shorter remission times and impaired survival, including t(4;14), t(14;16), t(14;20), gain(1q21) and del(17p)^7^.

Using data from the phase III clinical trial MRC Myeloma IX, we previously described sex differences in the presence of molecular lesions in patients’ myeloma cells at the time of diagnosis and also in patient outcomes^8^. This study enrolled 1970 patients with newly diagnosed MM and looked at the role of bisphosphonates (sodium clodronate or zolendronic acid) and thalidomide in myeloma treatment^9^. Patients were randomised between induction therapy with cyclophosphamide, vincristine, doxorubicin, and dexamethasone (CVAD) or cyclophosphamide, thalidomide and dexamethasone (CTD), followed by high-dose therapy plus autologous stem cell transplantation (ASCT) for fitter patients and melphalan-prednisone (MP) or attenuated CTD (CTDa) for less fit patients^9^. At second randomisation, patients were assigned to thalidomide maintenance therapy or no maintenance^9^. This study found that the molecular risk lesions t(4;14), t(14;16) and gain(1q) were more common in females^8^. Female sex was associated with inferior overall survival (OS), consistent with the increased frequency of high-risk lesions. However, the absence of any difference in progression-free survival (PFS) argued against this difference being driven by tumor biology alone^8^.

In this paper we use data from the successor phase III UK NCRI Myeloma XI trial to further evaluate the association of sex with the incidence of cytogenetic risk lesions present prior to treatment initiation and patient outcomes. This trial recruited 3894 newly diagnosed patients of all ages, with pathways for transplant-eligible (TE) and transplant-ineligible (TNE) patients, and patients received immunomodulatory agent-based induction and maintenance therapies (described in more detail in the methods section)^10^.

## Methods

### Trial outline

Myeloma XI is a phase III, open-label, randomized trial for newly diagnosed patients of all ages with pathways for both transplant-eligible (TE) and transplant-ineligible (TNE) patients^10^. A total of 3894 patients enrolled in the trial. There were 3 potential randomizations in the study. The induction randomization compared the triplet combination of cyclophosphamide, lenalidomide, and dexamethasone to a similar combination with thalidomide (CRD vs CTD)^10^. Patients who had a suboptimal response to induction therapy were then randomized to receive cyclophosphamide, bortezomib, dexamethasone (CVD) versus no CVD. TNE patients received attenuated doses of treatment (CTDa or CRDa). Eligible patients then underwent ASCT and in both pathways a maintenance randomization compared lenalidomide (+/-vorinostat) until disease progression versus observation^10^.

### Molecular analysis

A subset of 1610 patients had molecular data available. Molecular analysis was performed on purified myeloma cells from patients’ bone marrow biopsies taken prior to treatment initiation as described previously^10^. Adverse cytogenetic lesions were defined as t(4;14), t(14;16), t(14;20), del(17p), and gain(1q). Standard risk (SR) was defined as the absence of any of these lesions, high-risk (HiR) as one lesion present, and ultra-high-risk (UHiR) as >1 lesion present.

### Response assessment

Response was assessed based on the International Myeloma Working Group (IMWG) Uniform Response Criteria for Multiple Myeloma: CR = complete response, VGPR = very good partial response, PR = partial response, MR = minimal response, NC = no change, PD = progressive disease^11^.

### Statistical methods

This was a post hoc analysis. Baseline characteristics of males and females were compared using Fisher's exact test for categorical characteristics and the Wilcoxon-Mann-Whitney test for continuous characteristics, with p < 0.05 the level considered statistically significant. Outcomes, progression-free (PFS), and overall survival (OS) were compared using the log-rank test.

## Results

### Patient characteristics and molecular features

Of the 3894 patients enrolled in the trial, 2268 (58%) were male and 1626 (42%) were female, in keeping with the known sex disparity in MM presentation. The differences in patient characteristics, laboratory values, and treatments received between the two sexes were examined (Table 1). Significant differences in haemoglobin, platelet count, and renal function were identified between males and females as would be expected and has previously been described^12^, ^13^, ^14^. There was a small but statistically significant difference in bone marrow plasma cell percentage at initial randomization, suggesting a higher level of disease burden in females (plasma cell % ≥20% 45.2% of males vs 47.3% of females). There was no significant difference in International Staging System (ISS) stage at baseline or in the treatment later received by patients of each sex. Female patients were more likely to have the molecular risk lesions t(14;16) (1.8% of males vs 4.2% of females, p = 0.004) and del(17p) (7.4% of males vs 10.6% of females, p = 0.023). Females also had proportionately more HiR and UHiR disease (males SR 57%, HiR 33%, UHiR 9.8%; females SR 51%, HiR 35%, UHiR 13%, p = 0.026) (Table 2).

**Table 1:** Patient characteristics at initial randomization, intention to treat population. WHO = World Health Organization; ISS = International Staging System; CTD = cyclophosphamide, thalidomide, dexamethasone; CRD = lenalidomide, cyclophosphamide, dexamethasone; CTDa = attenuated CTD; CRDa = attenuated CRD.

	Males n = 2268 n (%)	Females n = 1626 n (%)	Total n = 3894 n (%)	p - value
**Age at initial randomization (years)**				
Median (range)	67.0 (28.0, 92.0)	68.0 (28.0, 89.0)	68.0 (28.0, 92.0)	0.195
**WHO performance status**				
0	804 (35.4%)	541 (33.3%)	1345 (34.5%)	0.482
1	872 (38.4%)	671 (41.3%)	1543 (39.6%)	
2	355 (15.7%)	244 (15.0%)	599 (15.4%)	
3	109 (4.8%)	79 (4.9%)	188 (4.8%)	
4	12 (0.5%)	9 (0.6%)	21 (0.5%)	
Not available	116 (5.1%)	82 (5.0%)	198 (5.1%)	
**Plasma cell % on bone marrow aspirate**				**0.037**
<20%	494 (21.8%)	309 (19.0%)	803 (20.6%)	
≥20%	1026 (45.2%)	769 (47.3%)	1795 (46.1%)	
Not available	748 (33.0%)	548 (33.7%)	1296 (33.3%)	
**Paraprotein type**				0.612
IgG	1411 (62.2%)	996 (61.3%)	2407 (61.8%)	
IgA	561 (24.7%)	396 (24.4%)	957 (24.6%)	
IgM	8 (0.4%)	4 (0.2%)	12 (0.3%)	
IgD	21 (0.9%)	11 (0.7%)	32 (0.8%)	
Light chain only	250 (11.0%)	207 (12.7%)	457 (11.7%)	
Non-secretor	13 (0.6%)	10 (0.6%)	23 (0.6%)	
Not available	4 (0.2%)	2 (0.1%)	6 (0.2%)	
**Light chain type**				0.230
Lambda	730 (32.2%)	554 (34.1%)	1284 (33.0%)	
Kappa	1512 (66.7%)	1056 (64.9%)	2568 (65.9%)	
Missing	26 (1.1%)	16 (1.0%)	42 (1.1%)	
**ISS**				0.783
Stage I	533 (23.5%)	397 (24.4%)	930 (23.9%)	
Stage II	889 (39.2%)	627 (38.6%)	1516 (38.9%)	
Stage III	669 (29.5%)	491 (30.2%)	1160 (29.8%)	
Not available	177 (7.8%)	111 (6.8%)	288 (7.4%)	
**Induction randomization treatment**				0.117
CTD	611 (26.9%)	410 (25.2%)	1021 (26.2%)	
CRD	610 (26.9%)	411 (25.3%)	1021 (26.2%)	
CTDa	536 (23.6%)	388 (23.9%)	924 (23.7%)	
CRDa	511 (22.5%)	417 (25.6%)	928 (23.8%)	
**Maintenance randomization treatment**				0.095
No maintenance	449 (19.8%)	268 (16.5%)	717 (18.4%)	
Lenalidomide maintenance	559 (24.6%)	344 (21.2%)	903 (23.2%)	
Lenalidomide and vorinostat maintenance	171 (7.5%)	136 (8.4%)	307 (7.9%)	
Not randomized	1089 (48.0%)	878 (53.9%)	1967 (50.5%)

**Table 2:** Patient molecular features, including presence of genetic lesions and disease risk status. SR = standard risk disease; HiR = high-risk disease; UHiR = ultra-high-risk disease.

Genetic lesion	Males (n = 962) n (%)	Females (n = 648) n (%)	p - value
t(4;14)	105 (11%)	78 (12%)	0.487
t(14;16)	17 (1.8%)	27 (4.2%)	**0.004**
t(14;20)	9 (0.9%)	7 (1.1%)	0.774
del(17p)	71 (7.4%)	69 (10.6%)	**0.023**
gain(1q)	312 (32%)	226 (35%)	0.308
**Risk status**			
SR	551 (57%)	333 (51%)	**0.026**
HiR	317 (33%)	229 (35%)	
UHiR	94 (9.8%)	86 (13%)	

### Response and survival outcomes by sex

**Figure 1:**
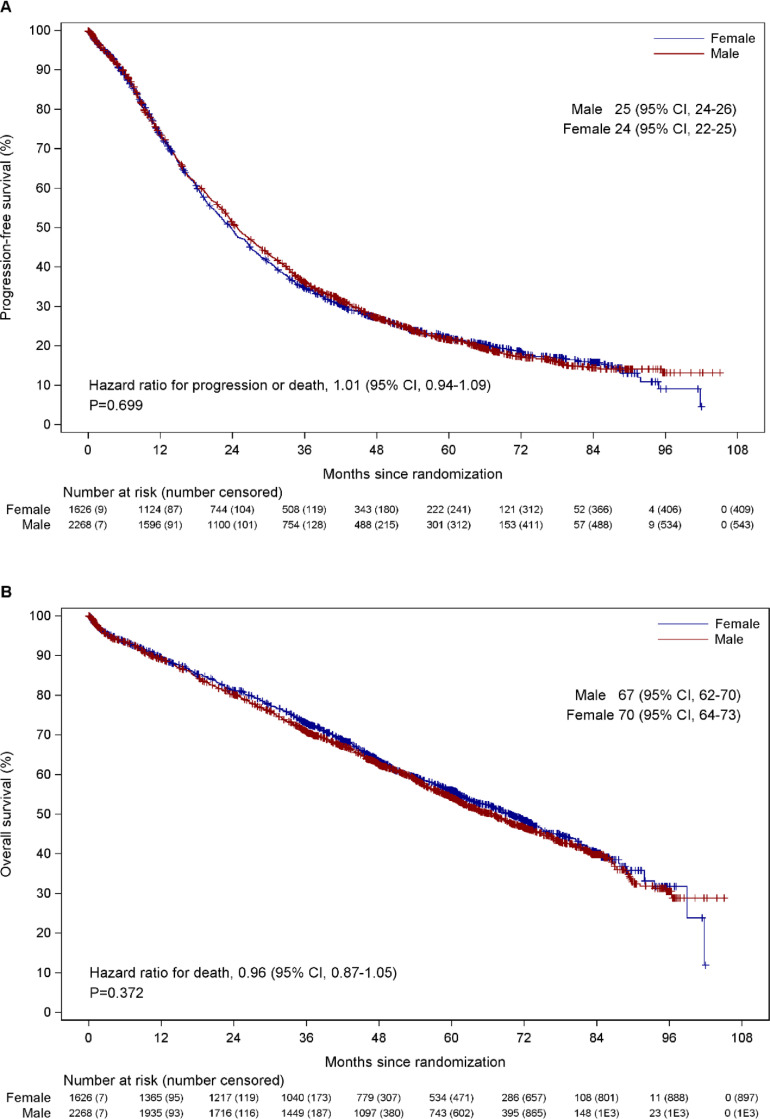
Overall PFS (A) and OS (B) for males and females from induction randomization. PFS = progression-free survival; OS = overall survival.

**Figure 2:**
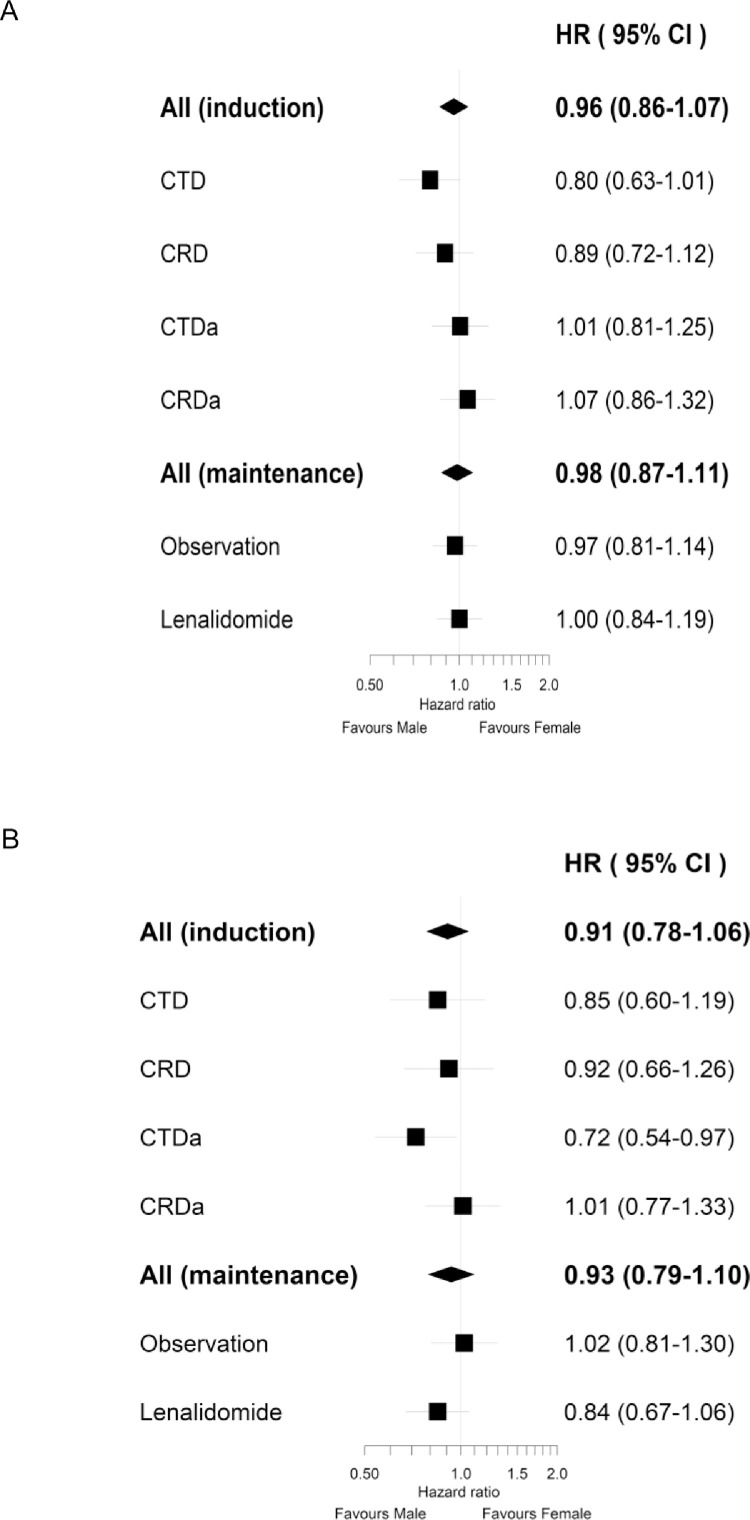
Forest plot of PFS (A) and OS (B) hazard ratios by induction regime and maintenance regime. PFS = progression-free survival; OS = overall survival; CTD = cyclophosphamide, thalidomide, dexamethasone; CRD = lenalidomide, cyclophosphamide, dexamethasone; CTDa = cyclophosphamide, thalidomide, dexamethasone (attenuated); CRDa = lenalidomide, cyclophosphamide, dexamethasone (attenuated).

Patient response at the end of induction chemotherapy was similar between males and females, both overall and within each treatment arm (CTD, CRD, CTDa, CRDa) (Supplementary Table 1). The overall response rate (≥PR) was 82.1% in males vs 80.3% in females and the percentage of patients achieving ≥VGPR was 54.4% in males vs 51.0% in females. PFS and OS from induction randomization did not significantly differ between males and females (Figures 1A-B). The PFS for males was 25 months (95% CI 24 - 26) and females was 24 months (95% CI 22 - 25), hazard ratio (HR) 1.01 (95% CI 0.94 - 1.09, p = 0.699). The OS for males was 67 months (95% CI 62 - 70) and females was 70 months (95% CI 64 - 73), HR 0.96 (95% CI 0.87 - 1.05, p = 0.372). There was no difference in PFS or OS between sexes when this was analyzed within the groups of patients randomized to receive different induction regimens or randomized to maintenance lenalidomide or observation (Figures 2A-B).

### Survival outcomes by sex and cytogenetic risk

**Figure 3:**
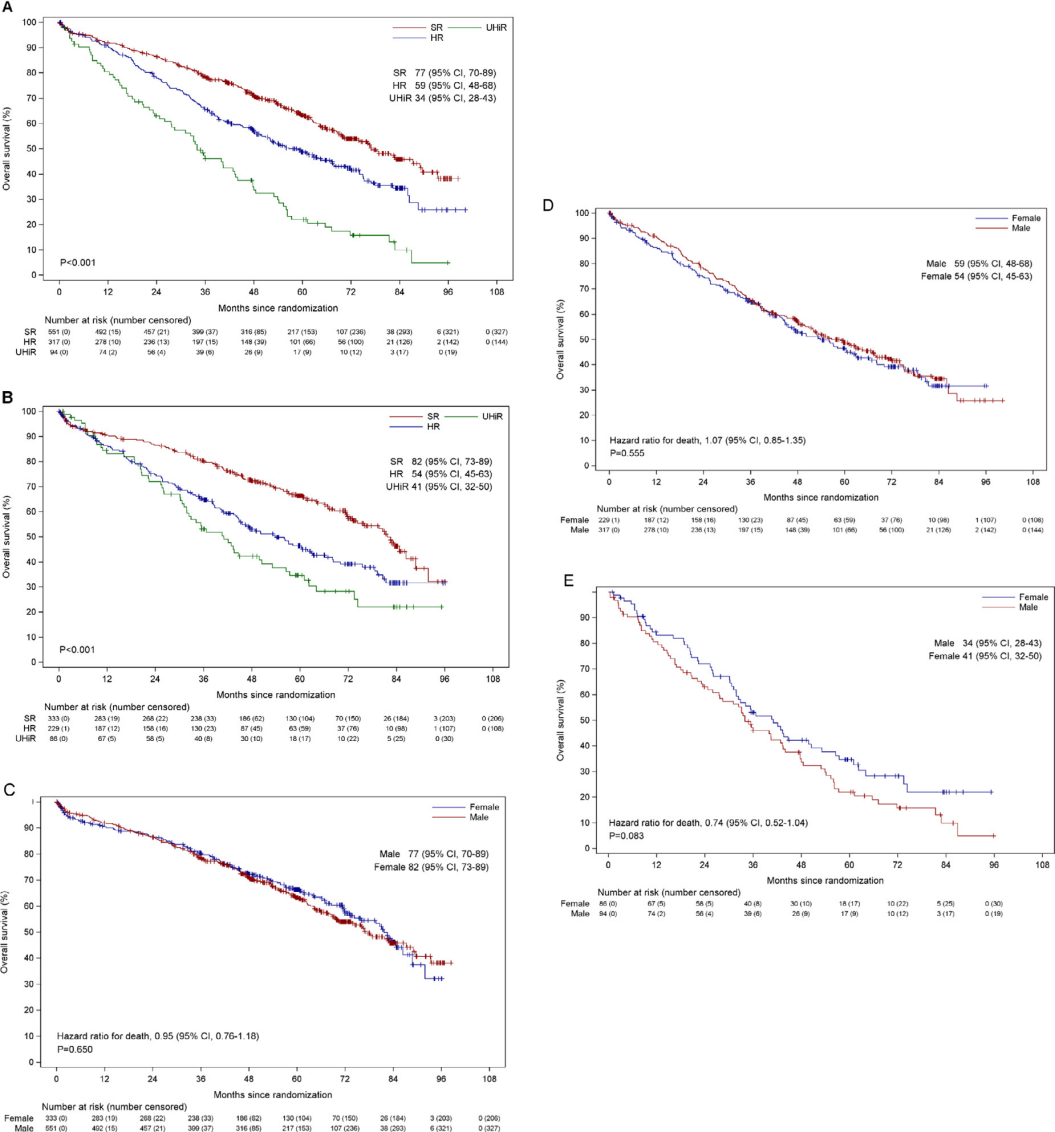
OS by risk status for males (A) and females (B). Comparison of OS for males and females with SR disease (C), HiR disease (D), and UHiR disease (E). OS = overall survival; SR = standard-risk disease; HiR = high-risk disease; UHiR = ultra-high-risk disease.

Molecular lesions that have been associated with outcome remained prognostic in both sexes, with a stepwise reduction in PFS and OS with cumulative risk lesions. Males with SR, HiR, and UHiR disease had a PFS of 29 months, 23 months, and 16 months respectively (p < 0.001) (Supplementary Figure 1A). For females with SR, HiR, and UHiR, the PFS was 27 months, 18 months and 17 months respectively (p < 0.001) (Supplementary Figure 1B). Males with SR, HiR, and UHiR disease had an OS of 77 months, 59 months, and 34 months respectively (p < 0.001) (Figure 3A). For females with SR, HiR, and UHiR, the OS was 82 months, 54 months, and 41 months respectively (p < 0.001) (Figure 3B).

Outcomes within each molecular risk category were compared between the sexes. There was no significant difference in PFS when we compared males and females in any molecular risk category (Supplementary Figures 1C-E). For OS (Figure 3 C-E) there was no difference in outcome in the SR or HiR groups, but in the UHiR group the OS for males was 34 months and for females 41 months, although this difference did not reach statistical significance, HR 0.74 (95% CI, 0.52-1.04, p = 0.083).

The difference in PFS and OS between sexes was next compared for each individual molecular risk lesion (Supplementary Figures 2A-F). In patients with del(17p), the PFS for males was 16 months and for females was 15 months, HR 1.09 (95% CI, 0.76-1.56, p = 0.648). For those with t(4;14), the PFS for males was 16 months and for females 18 months, HR 0.89 (95% CI 0.64-1.22, p = 0.456). For patients with t(14;16), the PFS for males was 16 months and for females 21 months, HR 0.85 (95% CI, 0.44-1.63, p = 0.624). In patients with del(17p), the OS for males was 28 months and for females 32 months, HR 0.98 (95% CI, 0.66-1.45, p = 0.913). For those with t(4;14), the OS for males was 45 months and for females 50 months, HR 0.86 (95% CI, 0.58-1.26, p = 0.438). For patients with t(14;16), the median OS for males and for females 35 months, although there appeared to be a later survival benefit in females, HR 0.60 (95% CI, 0.29-1.23, p = 0.161).

### Adverse events

Differences in the occurrence of adverse events between the two sexes were identified. Females were more likely to have a drop in neutrophil count during induction chemotherapy, however this did not appear to correspond to significantly higher risk of infection. In addition, females were more likely to suffer from diarrhoea, nausea, and vomiting, while males were more likely to suffer from myalgia (Supplementary Table 2).

## Discussion

The incidence of MM is higher in males as compared to females, suggesting that sex may affect aetiology and pathogenesis^8^. In this study female patients had a higher proportion of the adverse molecular risk lesions t(14;16), known to be a primary genetic event in MM, and del(17p), a secondary genetic event in MM. Furthermore, females were more likely to have HiR and UHiR disease. However, in the context of the Myeloma XI trial treatment, this did not correspond to a difference in PFS or OS, either overall or within each of the induction or maintenance randomization treatment options.

The difference in baseline genetic lesions identified in this study confirm some of the differences seen in our previous study, Myeloma IX^8^. Both the Myeloma IX and Myeloma XI analyses suggest that t(14;16) is significantly more common in females. In Myeloma IX, t(14;16) was present in 1.6% (10/644) of males and 5.7% (23/402) of females (p < 0.001), and in Myeloma XI it was present in 1.8% (17/962) of males and 4.2% (27/648) of females (p = 0.0038). We have therefore validated the t(14;16) translocation as a more common primary genetic event in females as compared to males. In Myeloma XI, del(17p) was found in 10.6% (69/648) of females and 7.4% of males (71/962), which was a statistically significant difference (p = 0.023). This difference had not been previously identified in the Myeloma IX data, with the lesion present in 7.6% of females (30/396) and 8.9% of males (55/618) (p = 0.489). This may be due to the higher numbers of patients in Myeloma XI, giving us more power to identify disparities. In the Myeloma XI data we did not find any difference between the sexes in the incidence of t(4;14) or gain(1q), both of which had been seen in Myeloma IX.

Sex differences in outcomes and the presence of various cytogenetic lesions have also been explored in other haematological malignancies. For example, in childhood and young-adult cases of acute myeloid leukaemia (AML), females have a significant survival advantage after controlling for other prognostic factors, HR 1.09 (95% CI, 1.00-1.18)^15^. This advantage was most clearly seen in patients aged 20-24 years, Caucasians, and AML subtypes AML-inv(16), acute promyelocytic leukaemia (which is characterised by t(15;17)), and acute erythroid leukaemia^15^. It has also been shown that there is sex disparity in the occurrence of chronic lymphocytic leukaemia (CLL), with a male-to-female ratio of 1.5^16^. One study used fluorescence in situ hybridization (FISH) to look for differences in genetic changes between the sexes in CLL and found that trisomy 12 and deletions of 11q22.3, 13q14.3, and 17p13.1 were more common in males. The team hypothesized that interactions between the autosomal abnormalities and sex chromosomes may provide the genetic basis for the excess of CLL cases in males^16^. However, as in MM, the biological processes driving these differences are still unclear.

*IGH* translocations in MM have been shown to result from at least 5 different mechanisms including class switch recombination (CSR), homologous recombination, somatic hypermutation, aberrant V(D)J rearrangement, and receptor revision^17^. The t(14;16) translocation is most frequently caused by CSR^17^. CSR is the process by which proliferating IgM-positive B cells rearrange the constant region genes in the *IGH* locus to switch from expressing IgM to IgG, IgE, or IgA, thereby producing an antibody with different effector properties but the same antigen specificity^18^. CSR is a multistep process that relies on the expression of activation-induced cytidine deaminase (AID)^18^. AID functions by deaminating cytidine which, coupled with base-excision repair or mismatch repair machinery, leads to the creation of mutations^19^. Interestingly, AID expression may be affected by sex hormones; oestrogen and progesterone have been associated with the modulation of AID expression in murine splenic B cells activated to undergo CSR^18^. When bound by oestrogen, the oestrogen-receptor induces AID transcription both directly, by binding and activating the AID promoter, and also indirectly, by binding and activating the *HoxC4* gene^18^. In contrast, the progesterone-bound progesterone receptor may inhibit AID transcription by binding upstream of the promoter^18^. Therefore, the regulation of AID by hormonal factors could possibly affect CSR mechanisms and perhaps form part of the explanation of sex disparities in certain translocations in MM.

In Myeloma IX, female sex was associated with inferior OS (median 49.9 months in males vs 44.8 months in females, p = 0.020) and no significant difference in PFS. In Myeloma XI there was no difference in PFS or OS and therefore we have not validated any difference in outcome. We hypothesized that this may have been due to the different therapy given in the Myeloma XI trial; however, there was no OS difference between the sexes in the Myeloma XI data for the CTD induction cohort and this regime was received by half of the patients in Myeloma IX. Other studies have identified either no difference in outcomes between the sexes^20^ or a worse outcome for male patients^21^^,^ ^22^. Posch et al performed a single-center study looking at sex-specific aspects in 191 patients with MM undergoing ASCT and found no difference in prognosis^20^. In contrast, in an analysis of ~3000 patients from 9 different clinical trials, males had a worse OS (HR for females 0.83, 95% CI, 0.75-0.91) and interstingly sex distribution was significantly different among different ethnic subgroups; 67.1% of Hispanics were male, 59.6% of non-Hispanic Whites, 51.8% of non-Hispanic African-Americans, and 45.4.% of non-Hispanic others (p = 0.002)^21^. In addition, a recent study by Derman et al explored outcomes by sex using two large population-based data sets (Surveillance, Epidemiology and End Results (SEER) data set and the Multiple Myeloma Research Foundation (MMRF) CoMMpass data set) and found that OS and PFS were improved for females. However, these patients did not have uniform treatment^22^.

Molecular risk stratifiers remained prognostic within both the male and female cohorts and there was a trend toward improved outcomes for females vs males within patients with t(14;16) and UHiR disease (but this did not reach statistical significance). The reasons behind the difference in OS sex disparity between the two analyses are unclear but could be related to a number of factors. For example, supportive care has changed in the period between the trials (Myeloma IX recruited 2003-2007, Myeloma XI recruited 2011-2017) and this could have affected males and females differently.

In conclusion, females were more likely to have the cytogenetic risk lesions t(14;16) and del(17p) and more HiR and UHiR disease. This was not associated with reduced PFS and OS, and therefore treatment in the context of the Myeloma XI trial might have been able to overcome some of the adverse effects of the risk lesions present.

## Clinical Practice Points

Females were more likely to have the poor prognosis lesions t(14;16) and del(17p), and were more likely to be assessed as having HiR or UHiR disease. However, this was not associated with reduced PFS or OS.

## Author contributions

SB, DAC, and CP designed this analysis; FED, GHJ, and GJM were Chief Investigators of the Myeloma XI trial; CP, KB, GC, MJ, JJ, MFK, RGO, GJM, GHJ, and FED participated in recruitment and management of patients; MFK, MTD, RGO, and GJM coordinated the central laboratory investigations; SB, DAC, TM, and CP analyzed and interpreted the data for this analysis; SB, DAC, and CP drafted the manuscript. All authors contributed to critically revising the manuscript and approved the final submitted version.

## Funding

Primary financial support for the Myeloma XI study was from Cancer Research UK [C1298/A10410]. Unrestricted educational grants from Celgene Corporation, Amgen, and Merck Sharp & Dohme, and funding from Myeloma UK supported trial coordination and laboratory studies. The authors are solely responsible for study design, data collection, data analysis and interpretation, writing, and decisions about publication submission; no funder had any role in these aspects of the trial. Trial data was accessible to all authors. SB is a National Institute of Health Research Academic Clinical Fellow. CP is a CRUK Clinician Scientist.

## Disclosures

**Sarah Bird:** No conflicts to declare. **David A. Cairns:** Celgene Corporation, Amgen, Merck Sharp & Dohme – research funding. **Faith E. Davies:** Adaptive – honoraria; Celgene Corporation – consultancy, honoraria, research funding; Janssen – consultancy, honoraria; Oncopeptide – consultancy, honoraria; Roche – consultancy, honoraria; Sanofi – consultancy, honoraria; Takeda – consultancy, honoraria. **Kevin Boyd:** Celgene, Janssen, Amgen – honoraria. Celgene, Janssen, Takeda, Novartis – consulting or advisory role. Celgene, Janssen, Takeda – travel support. **Tom Menzies:** Celgene Corporation, Amgen, Merck Sharp & Dohme – research funding. **Gordon Cook:** Takeda – consultancy, honoraria, research funding, speakers bureau; Glycomimetics – consultancy, honoraria; Sanofi – consultancy, honoraria, speakers bureau; Celgene Corporation – consultancy, honoraria, research funding, speakers bureau; Janssen – consultancy, honoraria, research funding, speakers bureau; Bristol-Myers Squibb – consultancy, honoraria; Amgen – consultancy, honoraria, research funding, speakers bureau. **Mark T. Drayson:** Abingdon Health – equity ownership, membership on an entity's board of directors or advisory committees. **Walter M. Gregory:** Celgene Corporation – consultancy, research funding; Amgen, Merck Sharp & Dohme – research funding; Janssen – honoraria. **Matthew W. Jenner:** Janssen – consultancy, honoraria, travel support, research funding; Takeda – consultancy, honoraria, travel support; Amgen – consultancy, honoraria, travel support; Celgene Corporation – consultancy, honoraria, research funding; Novartis – consultancy, honoraria. **John R. Jones:** Celgene Corporation – honoraria, research funding. **Martin F. Kaiser:** Bristol-Myers Squibb – consultancy, travel support; Chugai – consultancy; Janssen – consultancy, honoraria; Amgen – consultancy, honoraria; Takeda – consultancy, travel support; Celgene Corporation – consultancy, honoraria, research funding. **Roger G. Owen:** Takeda – honoraria, travel support; Janssen – consultancy, travel support; Celgene Corporation – consultancy, honoraria, research funding. **Graham H. Jackson:** Roche – consultancy, honoraria, speakers bureau; Amgen – consultancy, honoraria, speakers bureau; Janssen – consultancy, honoraria, speakers bureau; Merck Sharp & Dohme – consultancy, honoraria, speakers bureau; Celgene Corporation – consultancy, honoraria, travel support, research funding, speakers bureau; Takeda – consultancy, honoraria, travel support, research funding, speakers bureau. **Gareth J. Morgan:** Janssen – research funding; Bristol-Myers Squibb – consultancy, honoraria; Takeda – consultancy, honoraria; Celgene Corporation – consultancy, honoraria, research funding; Roche – consultancy, honoraria; Amgen – consultancy, honoraria; GSK – consultancy, honoraria; Karyopharm – consultancy, honoraria. **Charlotte Pawlyn:** Amgen – consultancy, travel support; Takeda Oncology – consultancy, travel support; Janssen – honoraria, travel support; Celgene Corporation – consultancy, honoraria, travel support.

## Data-sharing statement

De-identified participant data will be made available when all trial primary and secondary endpoints have been met. Any requests for trial data and supporting material (data dictionary, protocol, and statistical analysis plan) should be sent to ctru-dataaccess@leeds.ac.uk in the first instance. Only requests that have a methodologically sound proposal and whose proposed use of the data has been approved by the independent trial steering committee will be considered. Data requestors will need to sign a data access agreement.

## Appendix Supplementary materials

Image, application 1
