# Supplementary material for: Sex Differences in Multiple Myeloma Biology but not Clinical Outcomes: Results from 3894 Patients in the Myeloma XI Trial
Source: Clin Lymphoma Myeloma Leuk. 2021 Oct;21(10):667–75. doi: 10.1016/j.clml.2021.04.013 (PMC8528179; doi:10.1016/j.clml.2021.04.013)
Supplement: Supplementary file 1 [file mmc1.docx]

**Supplementary Table 1**

Response at the end of induction chemotherapy treatment. Statistical analysis considers the difference in proportion of males and females who achieve CR/VGPR. CR = complete response; CR (without BM) = results compatible with CR but without confirmation by bone marrow examinations; VGPR = very good partial response; PR = partial response; MR = minimal response; NC = no change; PD = progressive disease.

|  | **Males**  **n = 2268**  **n (%)** | **Females**  **n = 1626**  **n (%)** | **Total**  **n = 3894**  **n (%)** | **p - value** |
| --- | --- | --- | --- | --- |
| **Response at end of induction treatment** |  |  |  | **0.047** |
| CR or VGPR | 1233 (54.4%) | 829 (51.0%) | 2062 (53.0%) |  |
| CR | 149 (6.6%) | 96 (5.9%) | 245 (6.3%) |  |
| CR (without BM) | 583 (25.7%) | 416 (25.6%) | 999 (25.7%) |  |
| VGPR | 501 (22.1%) | 317 (19.5%) | 818 (21.0%) |  |
| PR or MR | 721 (31.8%) | 564 (34.7%) | 1285 (33.0%) |  |
| PR | 630 (27.8%) | 477 (29.3%) | 1107 (28.4%) |  |
| MR | 91 (4.0%) | 87 (5.4%) | 178 (4.6%) |  |
| NC or PD | 129 (5.7%) | 91 (5.6%) | 220 (5.6%) |  |
| NC | 44 (1.9%) | 33 (2.0%) | 77 (2.0%) |  |
| PD | 85 (3.7%) | 58 (3.6%) | 143 (3.7%) |  |
| Unable to assess | 52 (2.3%) | 36 (2.2%) | 88 (2.3%) |  |
| Death within 60 days of randomisation | 72 (3.2%) | 56 (3.4%) | 128 (3.3%) |  |
| Missing | 61 (2.7%) | 50 (3.1%) | 111 (2.9%) |  |

**Supplementary Figure 1**

PFS by risk status for males (A) and females (B). Comparison of PFS for males and females with SR disease (C), HiR disease (D), and UHiR disease (E). PFS = progression free survival; SR = standard risk disease; HiR = high-risk disease; UHiR = ultra-high-risk disease.


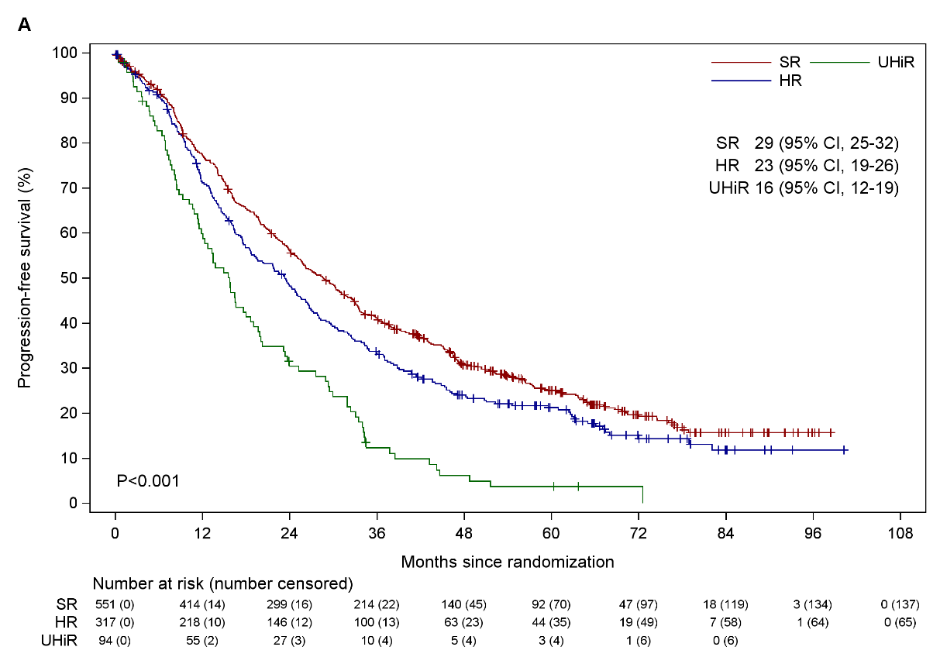


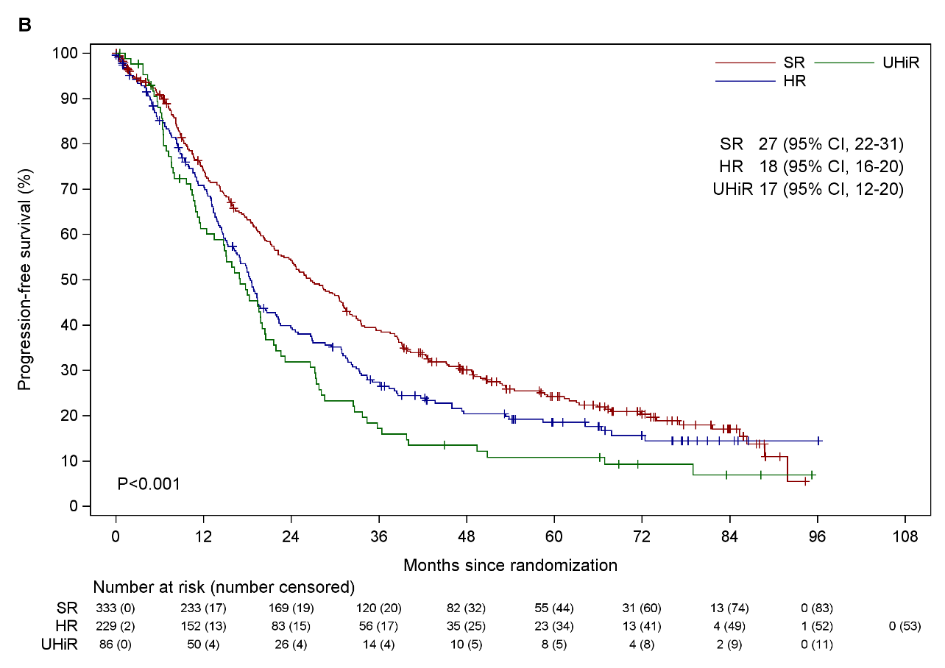


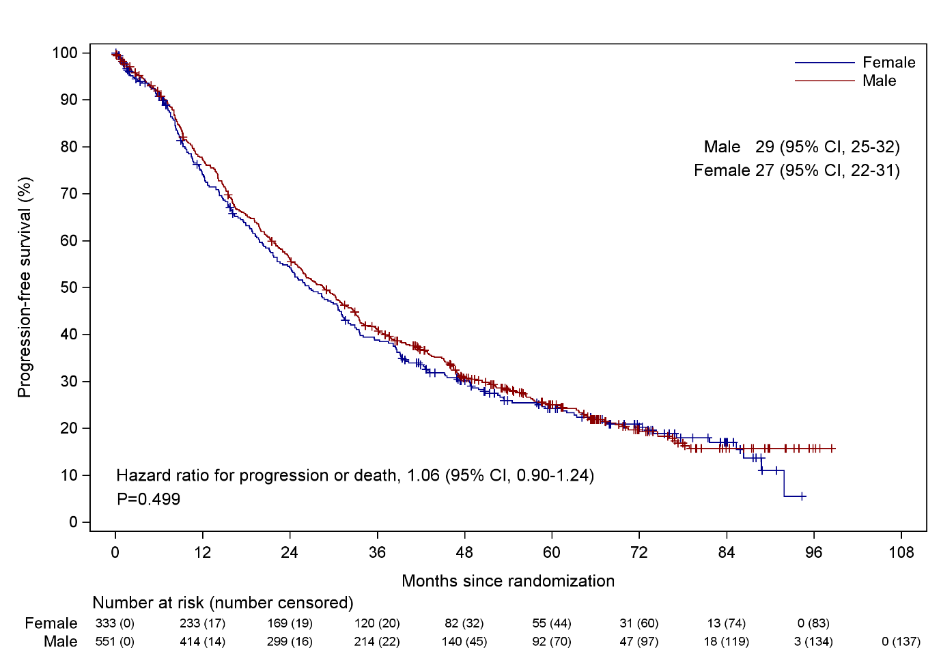


C


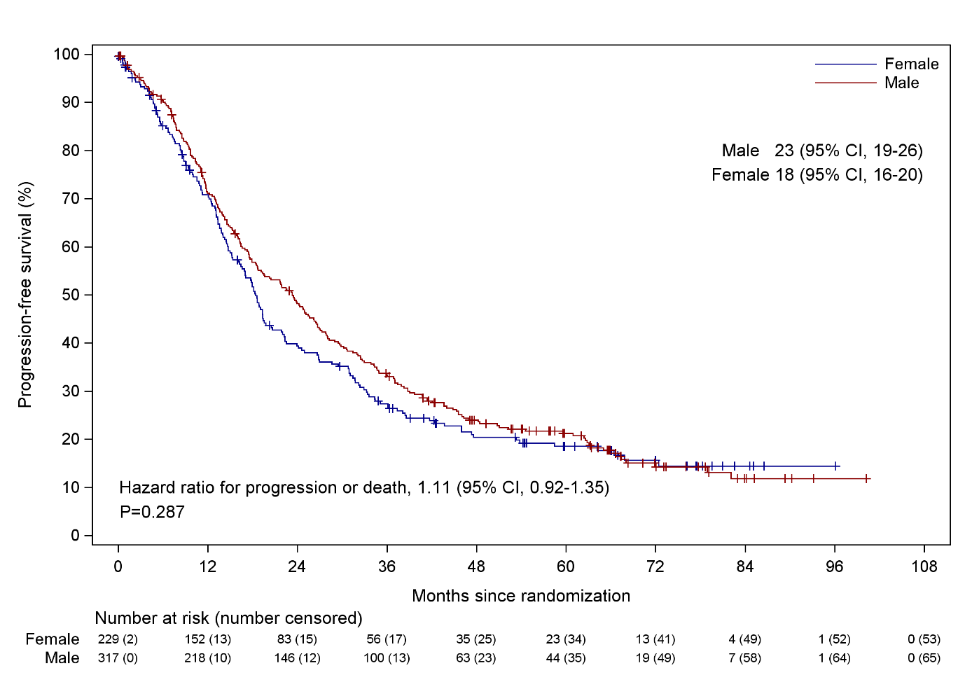


D


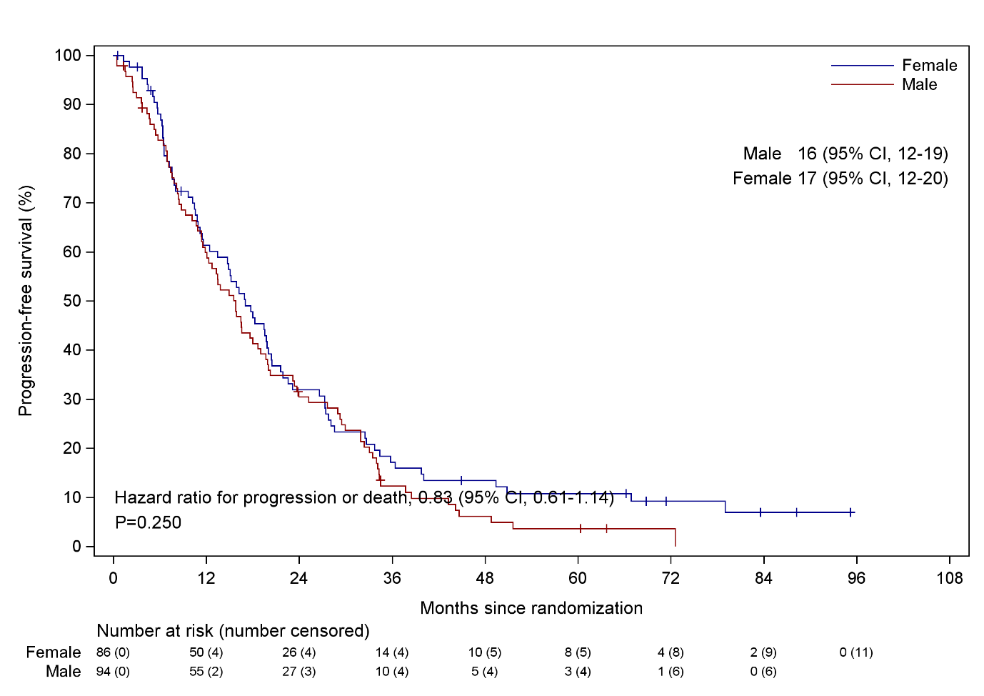


E

**Supplementary Figure 2**

Comparison of PFS for males and females with del(17p) (A), t(4;14) (B) and t(14;16) (C) and comparison of OS for males and females with del(17p) (D), t(4;14) (E) and t(14;16) (F). PFS = progression free survival; OS = overall survival.


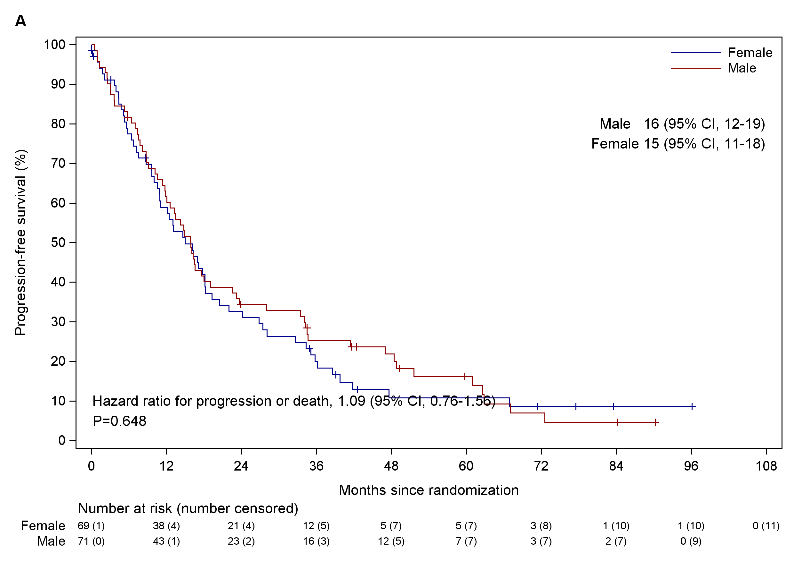


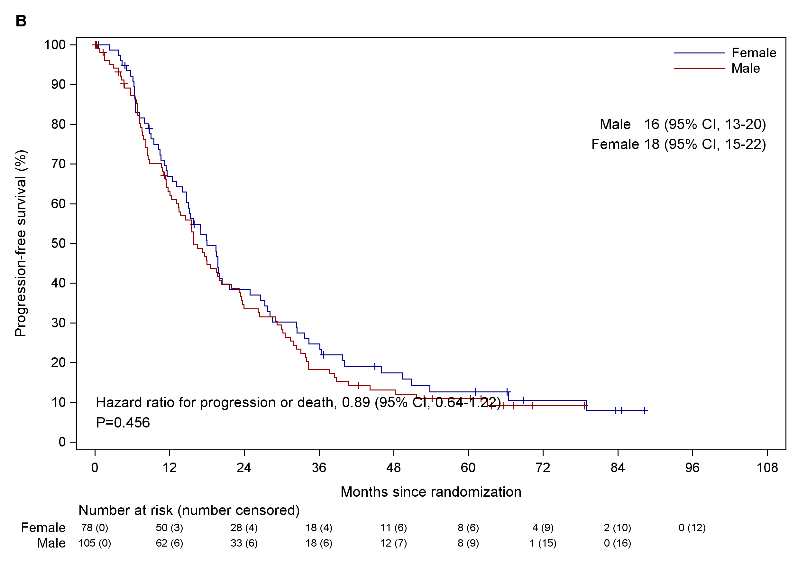


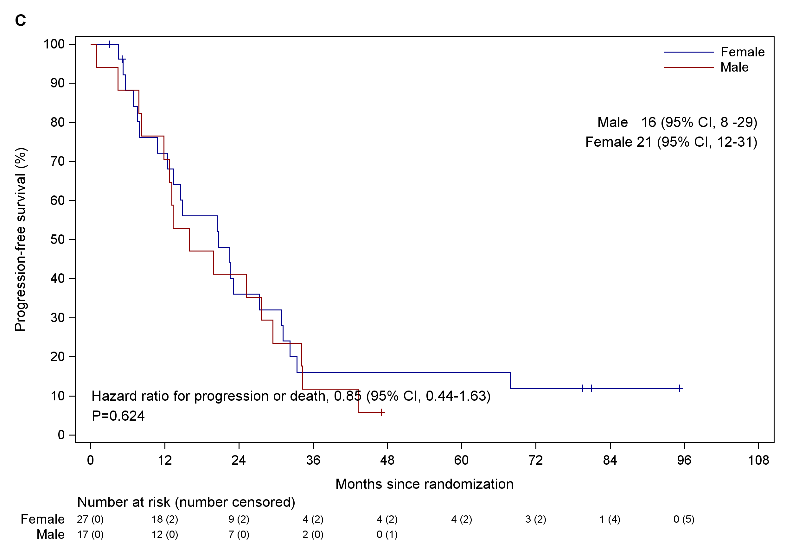


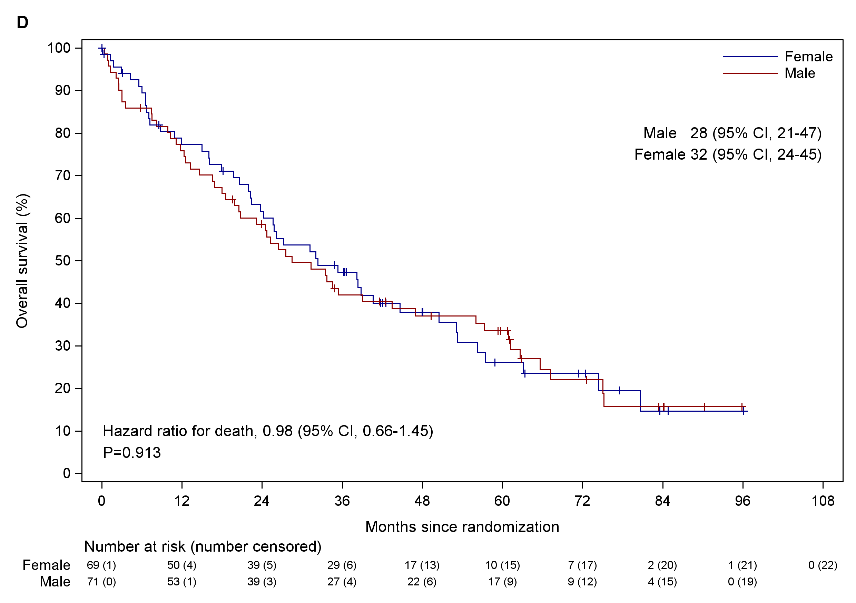


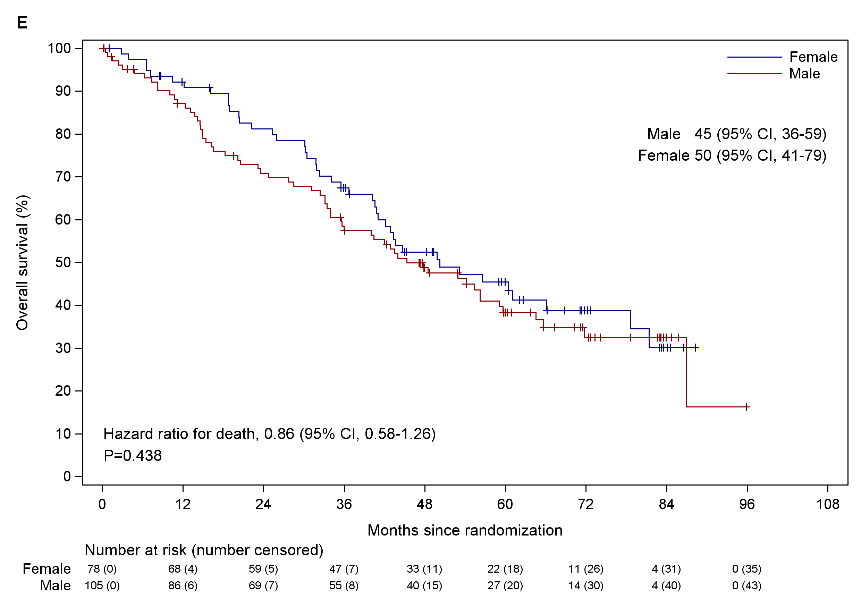


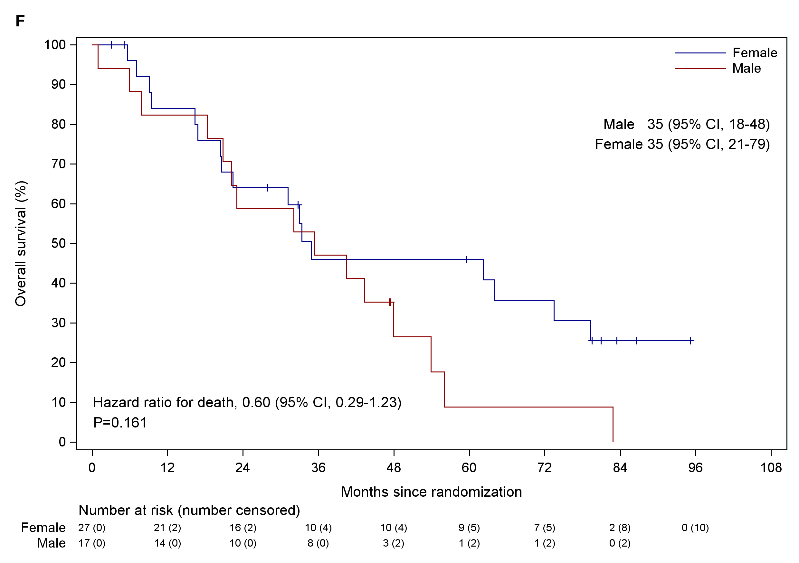


**Supplementary Table 2**

CTCAE grades, ordered alphabetically, of reported adverse reactions experienced during induction chemotherapy that were significantly different for males and females and occurred in ≥5% of patients (of either sex), safety population. CTCAE = Common Terminology Criteria for Adverse Events.

|  | **Male**  **n = 2242**  **n (%)** | **Female**  **n = 1606**  **n (%)** | **Total**  **n = 3848**  **n (%)** | **p - value** |
| --- | --- | --- | --- | --- |
| **Anaemia CTCAE Grade** |  |  |  | **<0.001** |
| Not experienced | 411 (18.3) | 337 (21) | 748 (19.4) |  |
| 1 | 939 (41.9) | 452 (28.1) | 1391 (36.1) |  |
| 2 | 660 (29.4) | 563 (35.1) | 1223 (31.8) |  |
| 3 | 184 (8.2) | 208 (13) | 392 (10.2) |  |
| 4 | 12 (0.5) | 18 (1.1) | 30 (0.8) |  |
| Missing | 36 (1.6) | 28 (1.7) | 64 (1.7) |  |
| **Constipation CTCAE Grade** |  |  |  | **0.027** |
| Not experienced | 957 (42.7) | 767 (47.8) | 1724 (44.8) |  |
| 1 | 705 (31.4) | 469 (29.2) | 1174 (30.5) |  |
| 2 | 511 (22.8) | 325 (20.2) | 836 (21.7) |  |
| 3 | 27 (1.2) | 14 (0.9) | 41 (1.1) |  |
| 4 | 6 (0.3) | 3 (0.2) | 9 (0.2) |  |
| Missing | 36 (1.6) | 28 (1.7) | 64 (1.7) |  |
| **Diarrhoea CTCAE Grade** |  |  |  | **<0.001** |
| Not experienced | 1850 (82.5) | 1221 (76) | 3071 (79.8) |  |
| 1 | 232 (10.3) | 208 (13) | 440 (11.4) |  |
| 2 | 89 (4) | 101 (6.3) | 190 (4.9) |  |
| 3 | 35 (1.6) | 45 (2.8) | 80 (2.1) |  |
| 4 | 0 (0) | 3 (0.2) | 3 (0.1) |  |
| Missing | 36 (1.6) | 28 (1.7) | 64 (1.7) |  |
| **Fatigue CTCAE Grade** |  |  |  | **0.018** |
| Not experienced | 1499 (66.9) | 1032 (64.3) | 2531 (65.8) |  |
| 1 | 399 (17.8) | 274 (17.1) | 673 (17.5) |  |
| 2 | 269 (12) | 221 (13.8) | 490 (12.7) |  |
| 3 | 37 (1.7) | 49 (3.1) | 86 (2.2) |  |
| 4 | 2 (0.1) | 2 (0.1) | 4 (0.1) |  |
| Missing | 36 (1.6) | 28 (1.7) | 64 (1.7) |  |
| **Mucositis oral**  **CTCAE**  **Grade** |  |  |  | **<0.001** |
| Not experienced | 2145 (95.7%) | 1497 (93.2%) | 3642 (94.6%) |  |
| 1 | 41 (1.8%) | 50 (3.1%) | 91 (2.4%) |  |
| 2 | 17 (0.8%) | 31 (1.9%) | 48 (1.2%) |  |
| 3 | 3 (0.1%) | 0 (0.0%) | 3 (0.1%) |  |
| Missing | 36 (1.6%) | 28 (1.7%) | 64 (1.7%) |  |
| **Myalgia CTCAE Grade** |  |  |  | **0.001** |
| Not experienced | 1950 (87) | 1458 (90.8) | 3408 (88.6) |  |
| 1 | 207 (9.2) | 94 (5.9) | 301 (7.8) |  |
| 2 | 45 (2) | 22 (1.4) | 67 (1.7) |  |
| 3 | 4 (0.2) | 4 (0.2) | 8 (0.2) |  |
| Missing | 36 (1.6) | 28 (1.7) | 64 (1.7) |  |
| **Nausea CTCAE Grade** |  |  |  | **<0.001** |
| Not experienced | 1856 (82.8) | 1165 (72.5) | 3021 (78.5) |  |
| 1 | 246 (11) | 265 (16.5) | 511 (13.3) |  |
| 2 | 89 (4) | 134 (8.3) | 223 (5.8) |  |
| 3 | 15 (0.7) | 14 (0.9) | 29 (0.8) |  |
| Missing | 36 (1.6) | 28 (1.7) | 64 (1.7) |  |
| **Neutrophil count decrease CTCAE Grade** |  |  |  | **<0.001** |
| Not experienced | 1091 (48.7) | 648 (40.3) | 1739 (45.2) |  |
| 1 | 362 (16.1) | 260 (16.2) | 622 (16.2) |  |
| 2 | 319 (14.2) | 245 (15.3) | 564 (14.7) |  |
| 3 | 312 (13.9) | 282 (17.6) | 594 (15.4) |  |
| 4 | 118 (5.3) | 141 (8.8) | 259 (6.7) |  |
| 5 | 4 (0.2) | 2 (0.1) | 6 (0.2) |  |
| Missing | 36 (1.6) | 28 (1.7) | 64 (1.7) |  |
| **Platelet count decrease CTCAE Grade** |  |  |  | **0.003** |
| Not experienced | 1513 (67.5) | 1145 (71.3) | 2658 (69.1) |  |
| 1 | 497 (22.2) | 285 (17.7) | 782 (20.3) |  |
| 2 | 106 (4.7) | 62 (3.9) | 168 (4.4) |  |
| 3 | 64 (2.9) | 57 (3.5) | 121 (3.1) |  |
| 4 | 25 (1.1) | 29 (1.8) | 54 (1.4) |  |
| 5 | 1 (0) | 0 (0) | 1 (0) |  |
| Missing | 36 (1.6) | 28 (1.7) | 64 (1.7) |  |
| **Tremor**  **CTCAE Grade** |  |  |  | **0.019** |
| Not experienced | 1930 (86.1) | 1339 (83.4) | 3269 (85.0) |  |
| 1 | 215 (9.6) | 170 (10.6) | 385 (10.0) |  |
| 2 | 56 (2.5) | 59 (3.7) | 115 (3.0) |  |
| 3 | 5 (0.2) | 10 (0.6) | 15 (0.4) |  |
| Missing | 36 (1.6) | 28 (1.7) | 64 (1.7) |  |
| **Urinary tract infection**  **CTCAE Grade** |  |  |  | **<0.001** |
| Not experienced | 2129 (95.0) | 1462 (91.0) | 3591 (93.3) |  |
| 1 | 7 (0.3) | 14 (0.9) | 21 (0.5) |  |
| 2 | 48 (2.1) | 83 (5.2) | 131 (3.4) |  |
| 3 | 21 (0.9) | 19 (1.2) | 40 (1.0) |  |
| 4 | 1 (0.0) | 0 (0.0) | 1 (0.0) |  |
| Missing | 36 (1.6) | 28 (1.7) | 64 (1.7) |  |
| **Vomiting CTCAE Grade** |  |  |  | **<0.001** |
| Not experienced | 2066 (92.1) | 1388 (86.4) | 3454 (89.8) |  |
| 1 | 86 (3.8) | 97 (6) | 183 (4.8) |  |
| 2 | 38 (1.7) | 60 (3.7) | 98 (2.5) |  |
| 3 | 16 (0.7) | 30 (1.9) | 46 (1.2) |  |
| 4 | 0 (0) | 3 (0.2) | 3 (0.1) |  |
| Missing | 36 (1.6) | 28 (1.7) | 64 (1.7) |  |
